# Life Course Theory, the Web of Violence, and Elder Mistreatment: Insights From the Better Together Couples’ Study

**DOI:** 10.1093/geroni/igaf122.1425

**Published:** 2025-12-31

**Authors:** Elizabeth Avent, Renee Garbe, Laura Mosqueda, Zachary Gassoumis

**Affiliations:** National Opinion Research Center at The University of Chicago, Chicago, Illinois, United States; The Pennsylvania State University, University Park, Illinois, United States; University of Southern California, Pasadena, California, United States; UTHealth Houston, Houston, Texas, United States

## Abstract

Elder mistreatment (EM) interventions often focus on mitigating immediate risk factors for perpetration and victimization, yet they frequently overlook the broader patterns of violence and abuse across the lifespan. Drawing on the web of violence framework and life course theory, this study underscores how EM is rarely an isolated occurrence but rather part of an interconnected trajectory of polyvictimization, intergenerational transmission of violence, and the co-occurrence of multiple forms of abuse over time. The Better Together Couples’ Study applies these theoretical frameworks to examine how early-life family violence and intimate partner violence (IPV) shape experiences of EM within dementia caregiving dyads. Qualitative interviews with caregivers revealed three key findings: (1) at least one partner in the dyad had a history of abuse, (2) past experiences of violence influenced their ability to recognize and define mistreatment, and (3) these histories shaped how they navigated caregiving for an abusive partner. These findings highlight the urgent need for EM prevention and intervention strategies that account for the long-term consequences of violence and abuse. Recognizing these patterns is critical for developing interventions that address the cumulative and relational nature of mistreatment across the life course. In addition to addressing immediate risk factors, interventions must incorporate a lifespan perspective, acknowledging how past trauma informs current caregiving dynamics and shapes vulnerability to mistreatment. Addressing these interconnections is essential for providing effective, trauma-informed support for older adults and their families with complex histories of abuse.

